# Profiling the circulating mRNA transcriptome in human liver disease

**DOI:** 10.18632/oncotarget.27617

**Published:** 2020-06-09

**Authors:** Aejaz Sayeed, Brielle E. Dalvano, David E. Kaplan, Usha Viswanathan, John Kulp, Alhaji H. Janneh, Lu-Yu Hwang, Adam Ertel, Cataldo Doria, Timothy Block

**Affiliations:** ^1^Baruch S. Blumberg Institute, Doylestown, PA, USA; ^2^University of Pennsylvania Perelman School of Medicine, Philadelphia, PA, USA; ^3^The Corporal Michael J. Crescenz Veterans Administration Hospital, Philadelphia, PA, USA; ^4^Current address: Department of Biochemistry and Molecular Biology, Medical University of South Carolina, Charleston, SC, USA; ^5^Department of Epidemiology, Human Genetics and Environment Science Center for Infectious Diseases School of Public Health, the University of Texas HSC, Houston, TX, USA; ^6^Department of Cancer Biology, Sidney Kimmel Cancer Center, Cancer Genomics Core Facility, Thomas Jefferson University, Philadelphia, PA, USA; ^7^Capital Health Cancer Center, One Capital Way, Pennington, NJ, USA

**Keywords:** hepatocellular carcinoma, circulating transcripts, biomarkers, liver cirrhosis, extracellular vesicles

## Abstract

The human circulation contains cell-free DNA and non-coding microRNA (miRNA). Less is known about the presence of messenger RNA (mRNA). This report profiles the human circulating mRNA transcriptome in people with liver cirrhosis (LC) and hepatocellular carcinoma (HCC) to determine whether mRNA analytes can be used as biomarkers of liver disease. Using RNAseq and RT-qPCR, we investigate circulating mRNA in plasma from HCC and LC patients and demonstrate detection of transcripts representing more than 19,000 different protein coding genes. Remarkably, the circulating mRNA expression levels were similar from person to person over the 21 individuals whose samples were analyzed by RNAseq. Liver derived circulating transcripts such as albumin (*ALB*), apolipoprotein (*APO*) A1, A2 & H, serpin A1 & E1, ferritin light chain (*FTL*) and fibrinogen like 1 (*FGL1*) were significantly upregulated in HCC patient samples. Higher levels of some of these liver-specific transcripts in the plasma of HCC patients were confirmed by RT-qPCR in another cohort of 20 individuals. Several less abundant circulating transcripts associated with cancer were detected in most HCC samples, but not in healthy subjects. Liver specificity of circulating transcripts was confirmed by investigating their expression in HCC tumor and liver cancer cell lines. Liver specific mRNA sequences in the plasma were predominantly present outside circulating extracellular vesicles.

Conclusions: The circulating “mRNA” transcriptome is remarkably consistent in diversity and expression from person to person. Detection of transcripts corresponding to disease selective polypeptides suggests the possibility that circulating mRNA can work as a biomarker analyte for cancer detection.

## INTRODUCTION

We are interested in using mRNA in the blood as a source of biomarkers of liver disease. Circulating non-coding RNA, such as microRNA (miRNA), is routinely detected in the blood [1–3]. However, detection and understanding of circulating mRNA sequences is in a much earlier research phase. Several laboratories have demonstrated the existence of circulating mRNA in healthy subjects and those with a disease diagnosis [3–13]. For example, circulating *hTERT* mRNA levels have been reported to correlate with a diagnosis of hepatocellular carcinoma (HCC) [14, 15] and with tumor progression [12, 16, 17]. Albumin (*ALB*) mRNA has also been detected in the circulation of advanced stage HCC patients and was reported to predict recurrence of HCC after transplantation [18, 19]. Additionally, during the preparation of this manuscript, circulating mRNA in cardiac disease patients was reported [3]. Although there are reports of specific mRNAs in select patient populations, a comprehensive study or “survey” specifically of circulating mRNA transcripts in liver disease patients has not been conducted. Circulating mRNA expression profiles, diversity, consistency, and heterogeneity across individuals is not clear. Also, the extent to which circulating mRNA is derived from “non-” blood tissue as opposed to blood or endothelial cells that line the blood is not clear. mRNA sequences in the blood, originating from non-blood cells, may be rare and/or may be representative of pathological events.

In this study, we analyzed total RNA isolated from adult human plasma. Briefly, RNAseq was performed on total RNA extracted from the plasma of 10 individuals with a diagnosis of HCC, 5 individuals with liver cirrhosis (LC) and no HCC, and 6 individuals with no evidence of liver disease. Seven of the 10 HCC individuals also had underlying LC. Protein coding transcripts, or mRNA, were readily detected in all plasma samples and liver selective mRNA transcripts were particularly prominent in the plasma from HCC/LC patients. Some of the circulating mRNA transcripts corresponded to genes whose expression is known to be altered or mutated in HCC. The presence of a selected set of liver specific mRNA transcripts in the circulation was confirmed by RT-qPCR analysis of another 20 distinct plasma samples.

Although we are ultimately interested in identifying circulating mRNA markers of liver disease, this study primarily intended to determine if there is a circulating mRNA transcriptome, to identify the extent of its diversity and consistency from sample to sample, and to lay the groundwork for methods that can be used to identify transcripts that correlate with a disease diagnosis. These parameters of the circulating human liver mRNA transcriptome are discussed.

## RESULTS

### Circulating RNA represents diverse transcripts from various tissues

**Figure 1:**
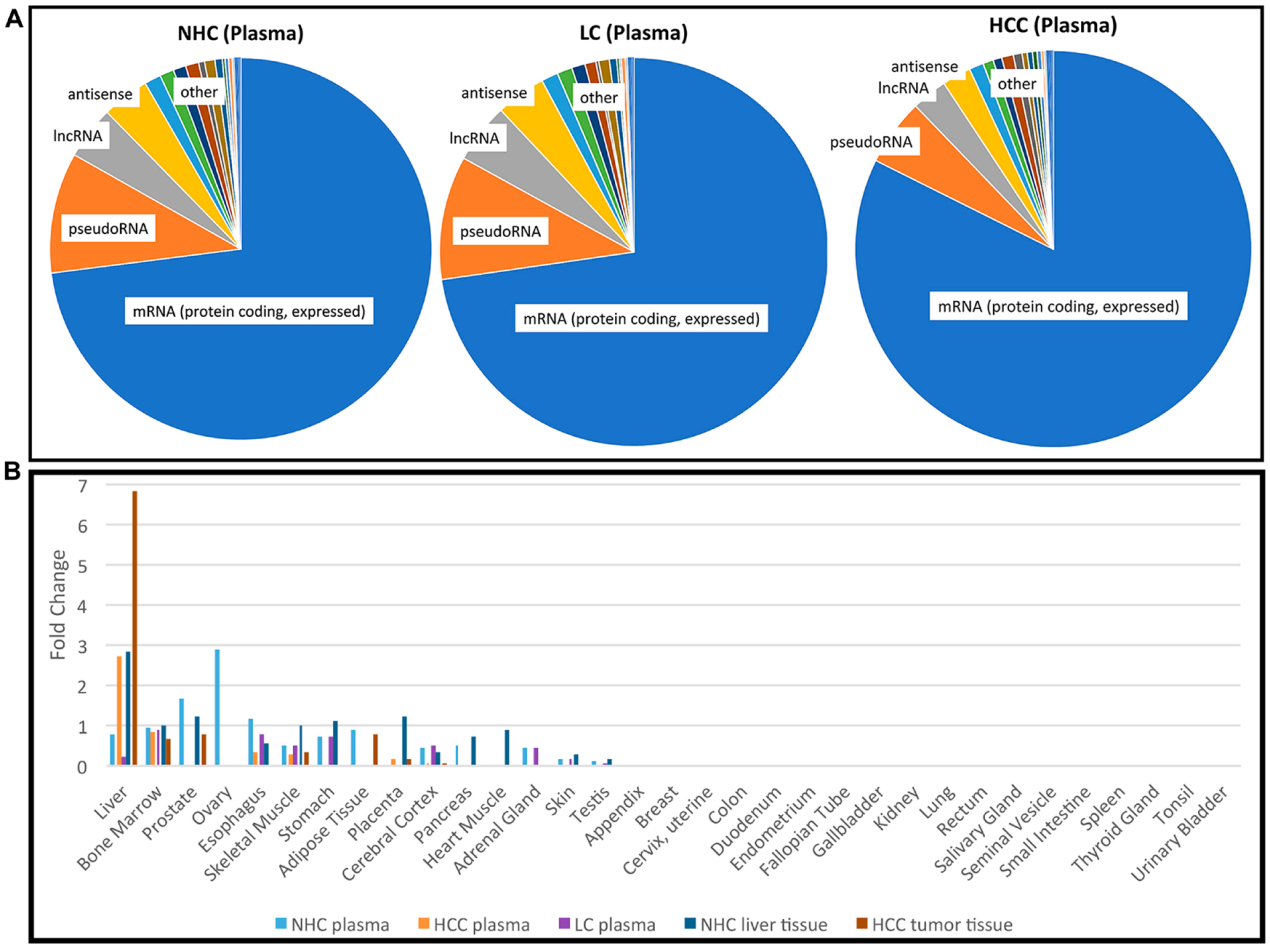
Profile of RNA present in the circulation. (**A**) Categories of the most abundant transcripts in the plasma from a cohort of HCC, LC, and NHC plasma samples. Pie charts represent the proportion of transcript types expressed in the top 25th percentile (75% to 100% most abundant) of HCC (*n* = 10), LC (*n* = 5), and NHC (*n* = 6) plasma samples. Charts were generated by first identifying the top 25% abundant transcripts and then counting how often each category appeared. (**B**) Tissues of origin of mRNA transcripts detected in human plasma. Five different sample sets were compared: NHC plasma (*n* = 6), HCC plasma (*n* = 10), LC plasma (*n* = 5), normal liver tissue (*n* = 1), and HCC tumor tissue (*n* = 2). The top most 1,200 abundant protein coding genes in each sample category were analyzed using TissueEnrich software (see Materials and Methods) and compared with tissue specific genes from publicly available RNAseq datasets (Human Protein Atlas and GTEx using algorithm [49]). Tissue enrichment is expressed as fold change in each category.

To evaluate the presence and diversity of mRNA transcripts in the human circulation, we performed RNAseq analysis on “total” cell-free RNA from the plasma of 10 individuals with HCC and LC, 5 with LC alone, and 6 “normal” healthy controls (NHC), bringing the total number of samples analyzed by RNAseq to 21 (Table 1). The HCC, LC, and healthy individuals were similar in age. Although both genders and those with HCC and LC with and without chronic viral hepatitis were included in all liver disease categories, balance was not achieved with this opportunistic set. For each sequenced sample, more than 50 million reads were generated and transcript abundances are reported as transcripts per million (TPM). Our methods were designed to reduce ribosomal RNA (rRNA) and mitochondrial RNA (mtRNA) (Supporting Information). Results of the circulating transcripts were first organized by abundance (sorted by highest to lowest TPM in normal plasma) and then highly abundant transcripts from the top quadrant/upper quartile were investigated for the frequency of each RNA category. Among the ~19,000 distinct mRNA transcripts found in circulation, 65–75% of the coding transcripts represented the top quartile of all circulating RNA isolated using our method and this result was consistent across different clinical categories (Figure 1A). However, when analysis was based upon the entire transcriptome (instead of just the 75–100% most abundant transcripts), mRNA represented only 15% of the identified circulating RNA (Supplementary Figure 1).

**Table 1:** Plasma samples used for RNAseq

	*n*	Age	Gender *n* = M/F	HBV/HCV/NAFL/NASH	LC (*n*)	HCC stages (*n*)	HCC Stages (*n*)
						AJCC I, II or BCLC A, B	AJCC III or BCLC C, D
NHC	6	60.0	3/3	0/0/0	N/A	N/A	N/A
LC	5	59.6	5/0	3/3/0	5	N/A	N/A
HCC	10	62.7	7/3	1/7/2	8	6	4

Early cancer (AJCC I, II; Barcelona A, B); Late (AJCC III; Barcelona C, D). N/A, Not Applicable. Plasma from 21 different individuals was used. *N* = 6, from “normal healthy controls” (NHC), *n **=*** 5 from those with a diagnosis of liver cirrhosis (LC) and *n **=*** 10 from those with a diagnosis of hepatocellular carcinoma (HCC) and LC. For the HCC group: 7 of the 10 were also diagnosed with LC, 2 with NAFL; 6 BCLC A, B or AJCC I, II; 2 BCLC C, D or 2 with AJCC III A & B. Ages and gender (Male [M], Female [F]), as indicated. Individuals with chronic hepatitis B virus (HBV) or hepatitis C virus (HCV) or no evidence of viral hepatitis were included, as indicated. Diagnosis of HCC, LC and chronic viral hepatitis as in text.

It was of interest to track the original tissue source/s of circulating coding transcripts. Therefore, RNAseq data from each of the HCC (*n* = 10), LC (*n* = 5), and NHC (*n* = 6) plasma samples, as well as HCC (*n* = 2) and normal liver (*n* = 1) tissues were analyzed using TissueEnrich software (see Materials and Methods). The most abundant 1,200 mRNA transcripts were investigated. As shown in Figure 1B, circulating mRNA in NHC subjects, which included samples from equal numbers of both genders, contained coding transcripts from a variety of organs (liver, bone marrow, stomach, esophagus, prostate). The plasma from those with HCC and, not surprisingly, normal liver and liver tumor tissue was greatly enriched for transcripts identified as liver derived. Of note is that plasma from the 5 individuals with LC (but no HCC) was reduced in liver derived transcripts. This result is curious since plasma from people with HCC was not reduced in liver derived transcripts, yet most (7 of 10) of those with HCC were also diagnosed with LC. This finding could be an artefact of the small sample size and not be representative of larger populations or perhaps, most likely, the presence of HCC influences liver transcript levels in the circulation. The apparent reduction in amount of female organ transcripts in the HCC samples reflects the male gender imbalance in this sample set. Taken together, these data show that the liver is a major source of coding RNA (mRNA) present in the circulation. The presence of liver derived mRNA transcripts in the circulation of HCC patients provides strong evidence that non-blood mRNA transcripts can be readily detected in circulation.

### Expression profiles of circulating mRNA transcripts show minimal variability between individuals

**Figure 2:**
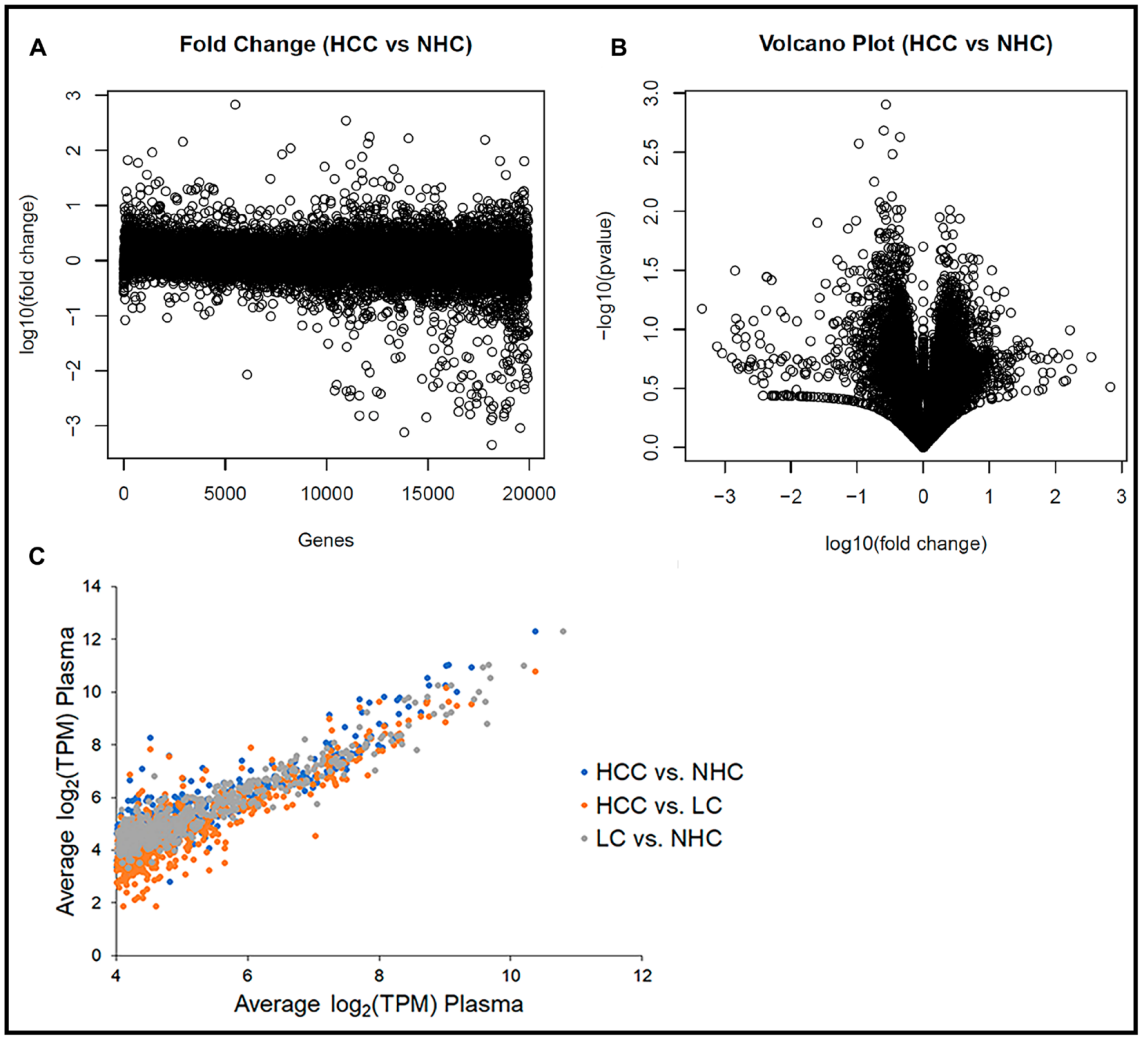
Human circulating transcriptome is essentially similar from person to person. (**A**) Transcript abundance (TPM) corresponding to each of ~19,000 coding regions in each sample as a function of fold difference from the mean of all samples. Plot of log_10_ (fold change) of the variation between HCC (*n* = 10) and NHC (*n* = 6) for each gene. (**B**) Volcano plot of the -log_10_ (fold change) of the variation between HCC (*n* = 10) and NHC (*n* = 6) vs. the *p*-value of the same variation. Genes toward the center of plot do not show significant variation, while those that are spread out show significant differences in terms of TPM. (**C**) The 1,000 most abundant circulating transcripts corresponding to protein coding genes graphed for each sample subset. Comparison of average gene expression (log_2_ (TPM)) of the top 1,000 transcripts between HCC vs. NHC, HCC vs. LC, and LC vs. NHC categories.

The consistency in levels (TPM) of any specific coding transcript between plasma samples was determined by calculating the fold change of a transcript between the HCC and NHC samples and between the LC and NHC samples. Figure 2A is a dot plot comparing log_10_ (fold change) between the HCC (*n* = 10) and NHC (*n* = 6) patient plasma and shows that more than 94% of the identified genes’ expression profiles varied less than 4 to 8-fold between the cohorts. Figure 2B shows a volcano plot of the -log_10_ (fold change) of the variation between HCC (*n* = 10) and NHC (*n* = 6) versus the *p*-value of the same variation, and also suggests that the human mRNA transcriptome does not vary significantly from person to person (genes centered in the plot), at least for the individuals of differing age, gender, and clinical condition analyzed here. Remarkably, the mRNA expression profile for each gene was generally consistent from person to person.

The consistency of expression profiles was also reflected in scatter plots with log_2_ (TPM) values for the 1,000 most abundant genes from each member of the NHC, HCC, and LC categories plotted against each gene’s average log_2_ (TPM) (data not shown). Consistent expression profiles were evident when the average log_2_ (TPM) values of each disease category were plotted against each other (Figure 2C). Most striking from this analysis, which includes all 21 plasma samples, was the consistency in transcript expression, for any given gene, from sample to sample. It is clear from the scatter plots that the closer a value plotted to a diagonal line (slope = 1), the closer it was to the average transcript amount, which indicated degrees of similarity or departure from the norm. It is noted that the highly abundant transcripts varied the least from patient to patient, the HCC patients had the greatest variability, and the variability was extreme for a subset of circulating transcripts.

These data collectively suggest that the circulating human transcriptome is relatively consistent from person to person, based on samples that range in age, gender, and liver disease category. That said, even though more than 94% of the gene transcripts between HCC vs. NHC and LC vs. NHC (data not shown) did not show more than 8-fold variation, there were some differences in mRNA expression patterns between HCC and healthy subject samples that offer the possibility of identifying disease selective biomarker transcripts.

### Liver derived mRNA sequences are among the most abundant transcripts in circulation

**Figure 3:**
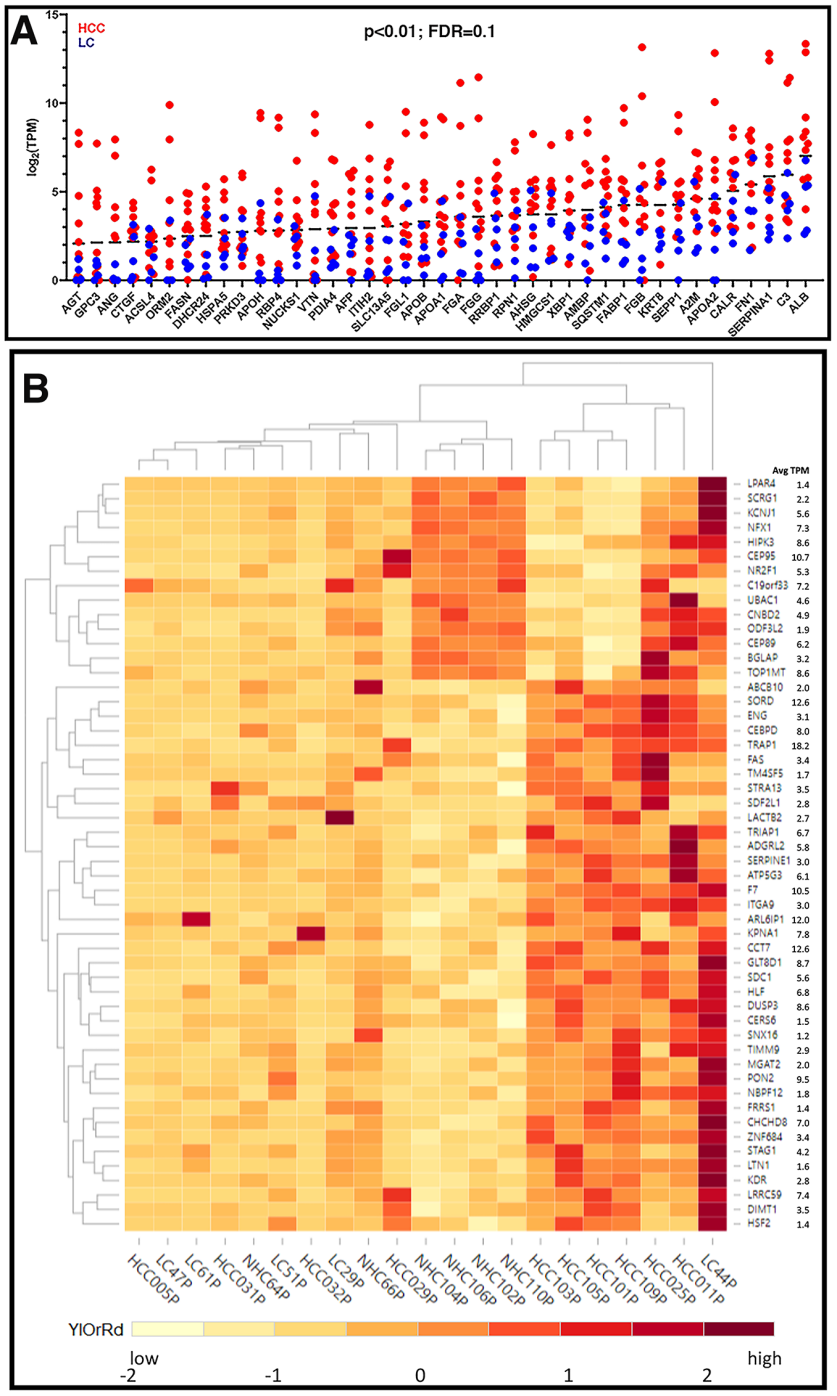
Expression profiles of unique circulating transcripts in liver disease. (**A**) Expression profiles (log2 (TPM)) of some abundant liver derived circulating transcripts with 3 to 7-fold higher expression in HCC samples compared to LC samples (*p* < 0.01). (**B**) Hierarchical clustering of differentially expressed genes. Differentially expressed transcripts were identified by sorting the normalized TPMs around the mean (zero) such that the scaled TPM values for HCC vs. NHC and LC vs. NHC are opposite in signs (negative, low expression and positive, high expression) and the difference between group averages was at least (± 1.5). The TPM values were scaled for each gene with the formula (x-μ)/σ, where x is variable data point, μ and σ are the average and standard deviation for a gene, respectively, with color scheme YlOrRd (yellow, orange, red).

Although most of the circulating coding transcripts did not vary significantly in expression from person to person (Figure 2), some features of the expression analysis are notable. The detection of mRNA transcripts in the plasma that correspond to polypeptides derived from or enriched in the liver, such as *ALB*, *APOA1*, *APOA2*, *APOB*, *APOH*, *SAA2*, *SERPINA1*, *SEPP1*, *ORM2*, etc. (Figure 3A), and from other non-blood cells was striking and provides evidence that the circulation contains mRNA that originated in the liver and not from artefacts of the phlebotomy process. These liver derived transcripts were upregulated in the circulation of HCC patients as compared to those with LC. Figure 3A represents expression patterns (log2 (TPM)) of some abundant (base mean TPM between 200 (*AGT*) and 9133 (*ALB*)) liver derived transcripts reflecting 3 to 7-fold higher levels compared to those of LC patients (*p* < 0.01).

A subset of 50 circulating transcripts was identified for their differential expression between HCC and NHC groups and between LC and NHC groups. Briefly, the expression levels of the 50 transcripts, relative to the mean level of expression of each transcript, was determined and is illustrated using a heat map (Figure 3B). Hierarchical clustering of the expression profiles in 21 plasma samples shows that the HCC samples clustered together in the heat map (Figure 3B). HCC samples did appear to selectively have a common subset of elevated levels of mRNA transcripts. Additionally, some circulating transcripts selectively upregulated in HCC samples correspond to genes associated with malignancy and, in many cases, HCC (e. g., *DUSP3* [20, 21], coagulation factor VII (*F7*) [22], *FAS* [23], *ITGA*9 [24, 25], *NBPF12* [26], *SNX16* [27], *SDF2L1* [28–30], *STRA13* [31], *TRAP1* [32], *SERPINE1* [33]). It is worth mentioning that the expression levels of 8 different transcripts corresponding to 5.8S rRNA were highly uniform in all samples analyzed by RNAseq, underscoring the accuracy and authenticity of RNAseq analysis and circulating expression profiles (data not shown). Despite using a method that aimed to minimize detection of mtRNA and rRNA, we observed that mitochondrial and ribosomal transcripts were the most highly abundant mRNA derived transcripts with uniform expression in the circulation of all individuals and could have been contributed by any organ or cells present in the blood (Supplementary Figure 2).

### Expression of circulating transcripts in HCC tumor and HCC cell lines

**Figure 4:**
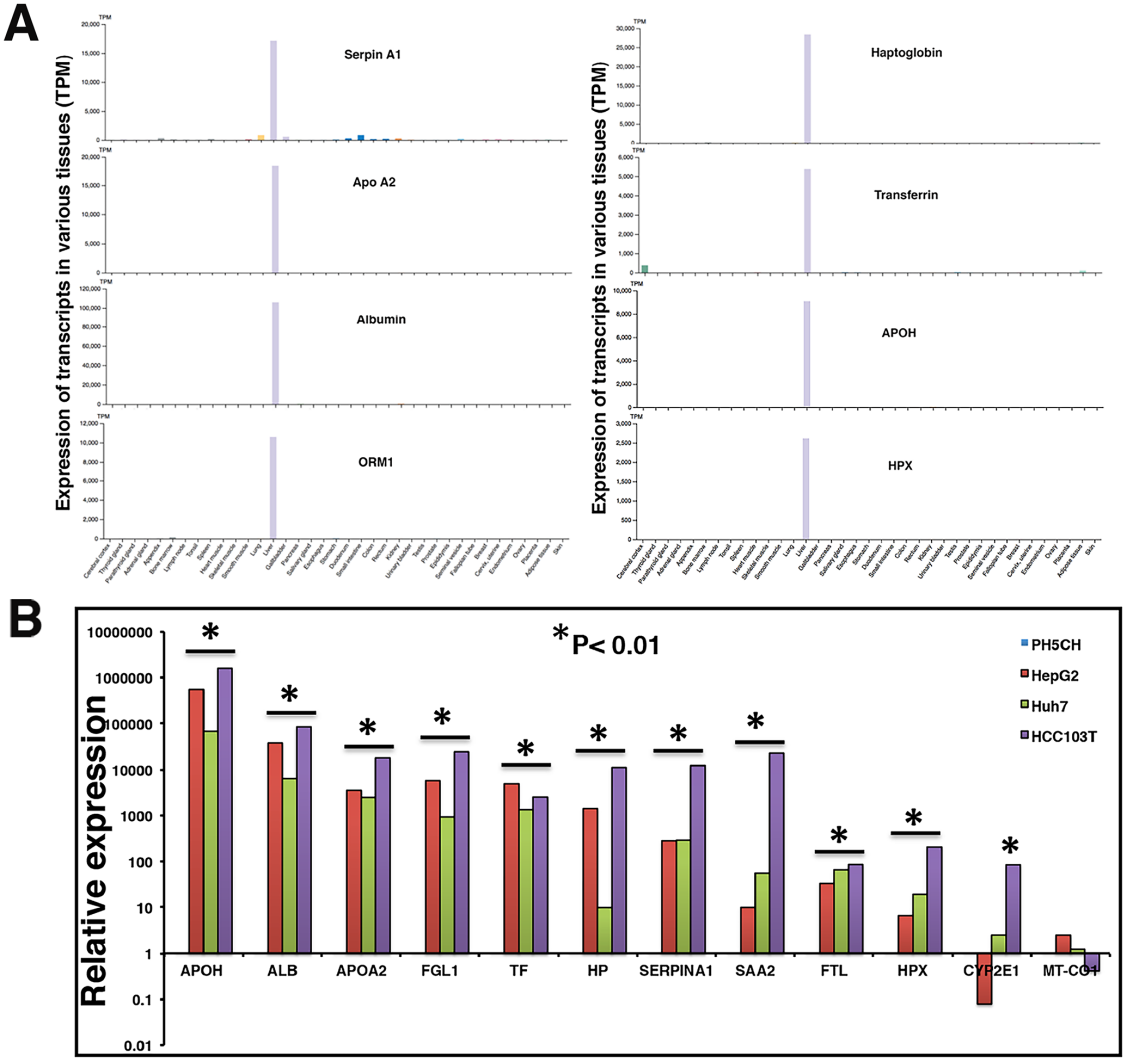
Liver specificity of circulating transcripts investigated in Human Protein Atlas (HPA) dataset, HCC tumor, and cell lines. (**A**) Expression profiles of transcripts were evaluated in HPA dataset (version 19.2, Ensemble version 92.38) representing expression levels in 37 human tissues based on RNAseq. Y-axis reflects expression in transcripts per million (TPM). (**B**) Relative expression of some liver specific transcripts was investigated in a tumor tissue (HCC103T) and cancer cell lines HepG2 and Huh7. Expression profiles were normalized with *GAPDH* expression and expression in non-malignant PH5CH liver cells. Each RT-qPCR reaction was carried out at least in triplicate and data are represented as mean values. Significant upregulation in comparison to control PH5CH cells is reflected by asterisk sign (*p* < 0.01).

Figure 1B above demonstrates that a substantial proportion of transcripts in the plasma of HCC patients are liver derived. Similarly, HCC tumor associated transcripts almost exclusively correspond to liver tissue. RNAseq data strongly suggest increased levels of a subset of liver specific transcripts in the circulation of HCC patients. Using the Human Protein Atlas (HPA) tissue gene expression dataset, which includes expression levels from 37 human tissues based on RNAseq, we identified a set of transcripts that are very selective for liver tissue, including *ALB*, *HP*, *APOA2*, *APOH*, *SERPINA1*, *ORM1*, and *HPX*. The genes’ extreme liver specificity is demonstrated in Figure 4A. The levels of these transcripts were then experimentally determined in a set of liver cancer cell lines and a moderately differentiated human tumor tissue (HCC103T) using RT-qPCR and are illustrated in Figure 4B. Expression levels were normalized with levels of corresponding transcripts in immortalized nonmalignant PH5CH liver cells. Our data demonstrate significant overexpression (*p* < 0.01) of these transcripts in tumor tissue, as well as cancer cell lines (Figure 4B).

### Validation and detection of circulating mRNA by RT-qPCR

**Figure 5:**
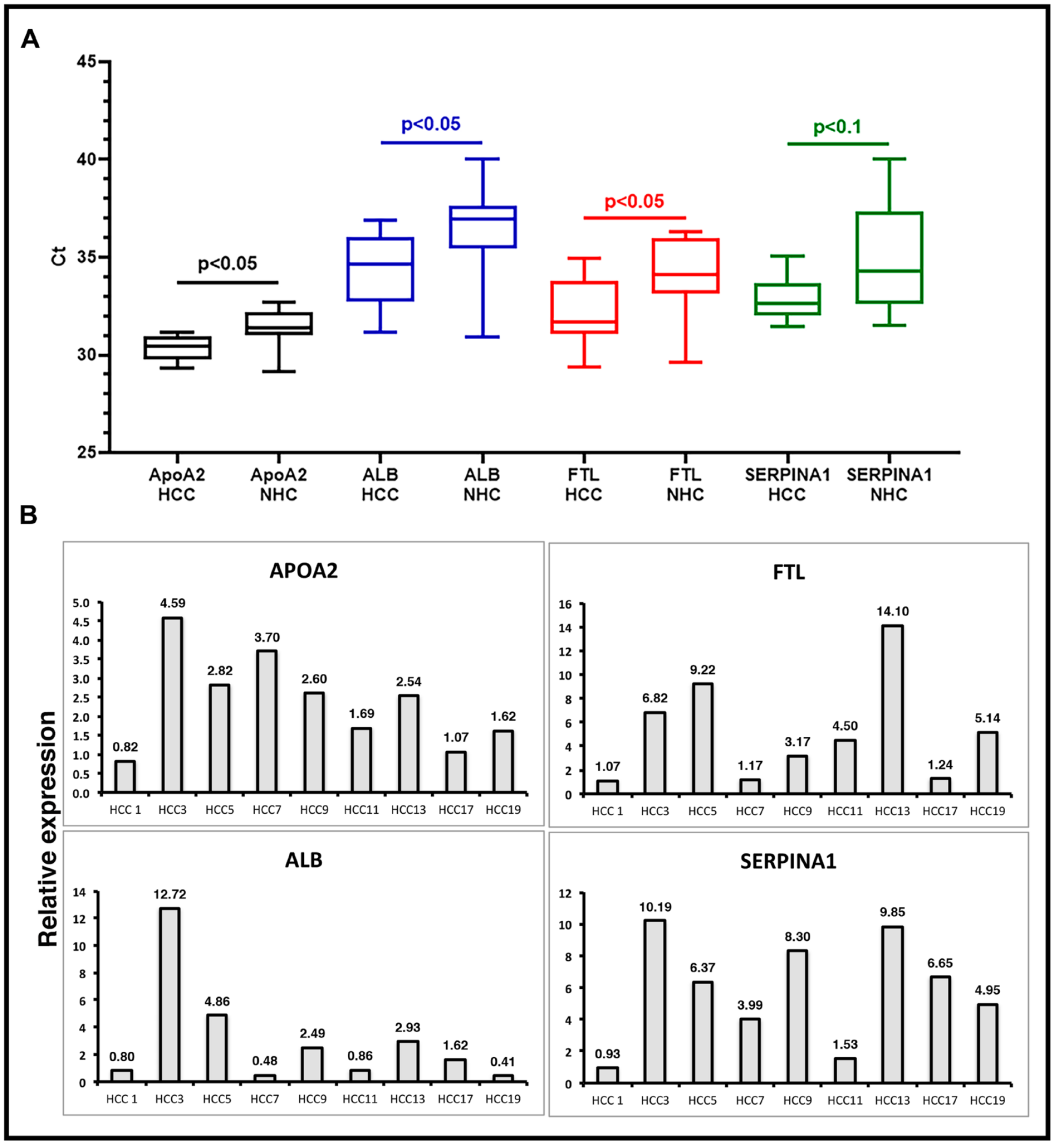
Validation of liver derived circulating transcripts by RT-qPCR in an independent dataset. (**A**) Relative expression of liver specific circulating transcripts in a cohort of 10 HCC patients compared to 10 age/gender matched normal healthy control (NHC) samples was investigated by RT-qPCR. Average Ct values in HCC and NHC samples are plotted. Statistical significance was calculated by unpaired Student’s *t*-test. (**B**) Relative expression calculated by ΔΔCt method. ΔCt values of HCC samples were calculated by subtracting spike-in ultramer Ct from Ct of target transcripts. Ct values of 10 NHC samples were averaged and normalized with respective ultramer Ct values to get an average NHC ΔCt. ΔΔCt of HCC samples was calculated by subtracting average NHC ΔCt from each HCC sample ΔCt. Relative expression calculated by formula 2^-ΔΔCt^ plotted as bar graphs. Each qPCR reaction was run at least in triplicate and data are represented as mean values. Sequence of oligos used can be found in Supplementary Materials and Methods.

To validate the presence of specific transcripts in patient circulation, we investigated a set of clinical samples that were studied earlier by RNAseq. RT-qPCR assays were carried out on circulating RNA from HCC patients (*n* = 4) and age and gender matched healthy controls (*n* = 4), and expression profiles of six liver derived transcripts were studied (Supplementary Figure 3). Data showed consistent expression profiles using RNAseq and RT-qPCR. Validation of circulating transcripts allowed for testing of specific mRNA transcripts in an additional independent set of samples from 20 individuals (10 with a diagnosis of HCC and 10 age and gender matched healthy subjects). In these 20 samples, the prevalence of 4 circulating liver specific transcripts, *FTL*, *APOA2*, *SERPINA1,* and *ALB*, was examined by RT-qPCR using exon specific primers (Supplementary Table 1). Figure 5A shows the results as average cycle threshold (Ct) values in samples from the HCC patients and NHCs. Ct values reflect the number of PCR cycles required for the fluorescent signal from the amplified product to cross a threshold or background. Primers specific for exons generated detectable products relatively consistent in amount from patient to patient. However, consistent with RNAseq data from the previous cohort, the transcripts tested were generally significantly upregulated in HCC samples as compared to the healthy subjects. Relative expression in HCC patients was calculated by normalizing with the Ct values of an internal reference control and also those of age and gender matched NHCs (Figure 5B). Figure 5B represents the same data as relative expression of *APOA2*, *ALB*, *FTL*, and *SERPINA1* transcripts in each HCC patient sample in comparison to the average amount present in the NHCs (*n* = 10). Again, although the number of patient and healthy subject samples analyzed are too small to draw conclusions, the trends of elevated levels of these transcripts in HCC samples relative to healthy subjects observed with the sample sets used in RNAseq does seem to be sustained. Indeed, some HCC samples had 9 to 20-fold elevations in liver specific transcripts compared to LC samples.

### Liver derived circulating transcripts predominantly map to genomic exons and represent mature mRNA

**Figure 6:**
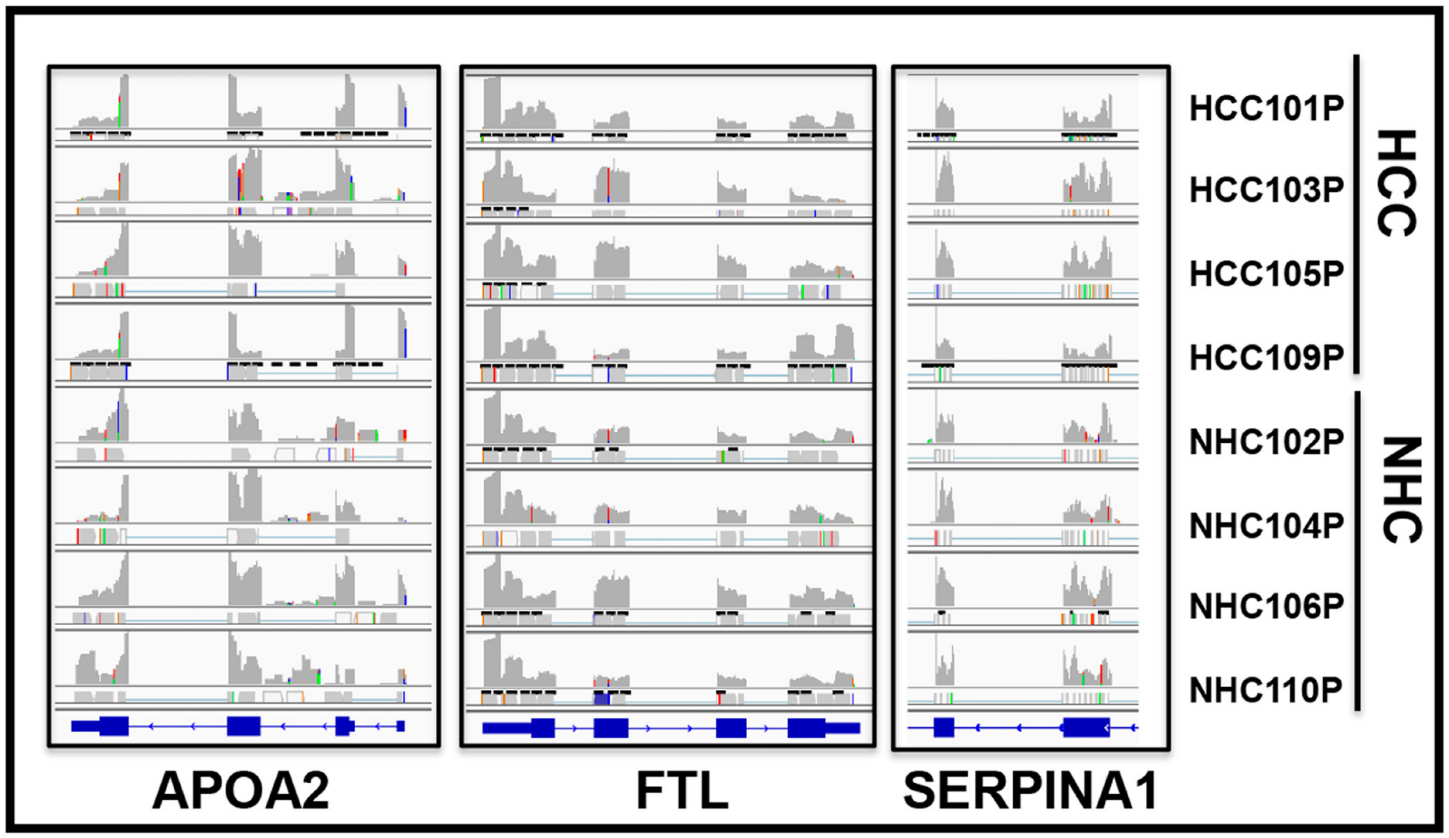
Circulating transcripts predominantly map to genomic exons. RNAseq read piles in plasma samples from 4 HCC patients and their age and gender matched normal healthy controls (NHC) analyzed in a single flow cell experiment. Alignment to 3 genes in 8 samples is shown and suggests that circulating transcript sequences overwhelmingly align to exons and not introns. Histograms show relative distribution of detected mRNA reads. Specific regions within exons show higher sequencing depth than others and patterns are largely consistent across samples. RefSeq gene model diagrams shown at bottom of read piles.

**Figure 7:**
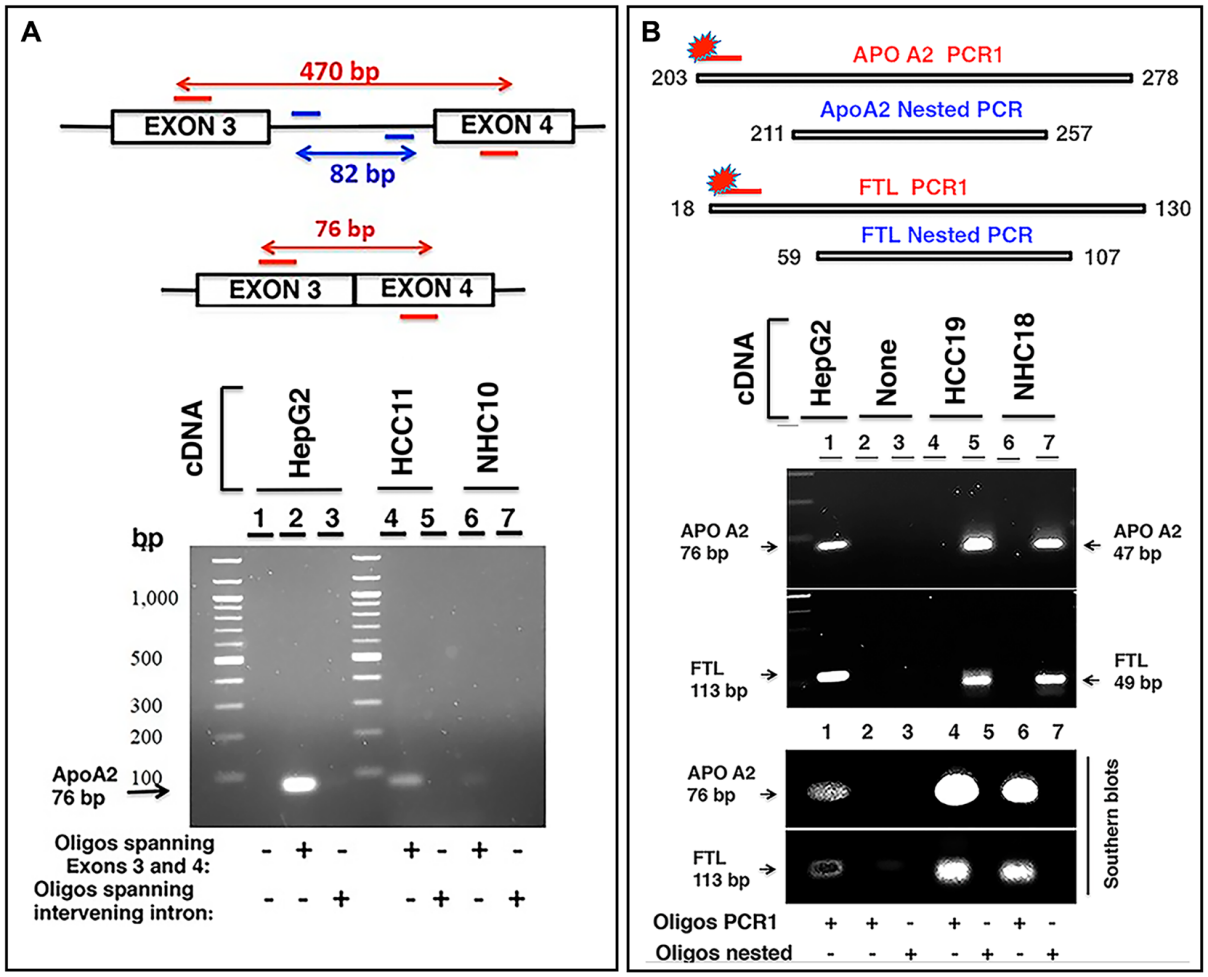
Characterization of circulating transcripts using semi-quantitative nested PCR and Southern blotting. (**A**) *APOA2* gene consisting of 4 exons was studied in two clinical samples, HCC11 (cancer patient) and NHC10 (normal healthy control) using 100 ng cDNA in each reaction. Two oligo sets were prepared: one set encompassing regions of exon 3 and exon 4 and the other set corresponding to the intervening intron (amplifying an 82 bp fragment). A short product of 76 bp was expected to be amplified by the former oligo set if no intron was present between the exons in the amplified fragment. Genomic DNA or even unspliced mRNA would yield a 470 bp product. Visualized gel demonstrates that the 76 bp *APOA2* product corresponding to primers spanning exons 3 and 4 is detected and shows higher expression in HCC11 sample compared to NHC10, but intron specific primers do not yield any product. HepG2 cDNA was used as a positive control. (**B**) Two sets of primer pairs, corresponding to PCR1 extended amplicon and nested amplicon were used to amplify *APOA2* and *FTL* transcripts in two samples, HCC19 and NHC18. PCR1 was carried out for 30 cycles using extended PCR1 oligos. PCR1 product was diluted 12.5-fold and subjected to nested PCR using nested oligos. Products were separated by electrophoresis on a 2% agarose gel. PCR1 products could not be detected, but the nested *APOA2* (47 bp) and *FTL* (49 bp) products were clearly observed. HepG2 cDNA was used as a control. As expected, the inner nested oligo sets yielded smaller products (47 bp for *APOA2*, 49 bp for *FTL*), while outer PCR1 sets of oligos yielded a larger product (76 bp for *APOA2*, 113 bp for *FTL*). Gel resolved PCR1 and nested PCR products were transferred to a nylon membrane followed by hybridization with extended PCR1 specific 5′-LICOR 800 tagged oligos. As expected, 5′-LICOR 800 probes did not detect the nested products. The positive control in lane 1, using PCR1 extended primer set and HepG2 cellular cDNA as template, reflects the extended PCR1 products of these transcripts. Lanes 2 and 3 are no template negative controls with either extended PCR1 (lane 2) or nested (lane 3) oligos. Lanes 4 and 6 reflect the extended PCR1 products in two patients, while lanes 5 and 7 represent nested products that are not detected by Southern blot using 5′-LICOR 800 tagged oligos in the same samples. Sequences of oligos can be found in Supplementary Materials and Methods.

If the RNA sequences detected in the circulation were derived from mRNA, the transcripts should align with genic exons and lack intronic sequences as introns are not present in most mature mRNA. We analyzed the read piles for 6 liver derived transcripts from at least 10 individuals (5 HCC, 5 NHC) and found substantial representation of exons in sequencing reads. As an example, Figure 6 shows read pile profiles of three circulating mRNA transcripts (*APOA2*, *FTL*, and *SERPINA1*) in 8 distinct plasma samples. The data show that the sequencing reads of circulating transcripts predominantly correspond to exonic regions and that certain specific regions within exons are detected at a higher sequencing depth than other regions within a given transcript. To confirm the selective detection of exons in liver derived transcripts, nested semi-quantitative PCR was performed on total, cell-free, DNase-treated RNA isolated from patient samples. Primers for *APOA2* that amplify either intronic or exonic sequences (that span the intron) were used. Our data show the detection of exon specific products alone without any intron specific or intron containing PCR products (Figure 7A). Further, detection of *APOA2* and *FTL* transcripts in patient plasma samples was also validated by nested PCR and LICOR 800 probe based Southern blotting (Figure 7B, Supplementary Table 1). The sensitivity of amplicon product detection increased when mRNA was either amplified using nested PCR or hybridized to gene specific probes via Southern blot (Figure 7B lower panel). Detection of a set of liver derived circulating transcripts in HCC plasma samples was also demonstrated using nCounter Elements assay (Nanostring technology), which directly measures mRNA transcripts without the need for reverse transcription and PCR amplification (data not shown).

### Liver derived transcripts predominantly detected outside extracellular vesicles (EVs) in circulation

Extracellular vesicles (EVs) consisting of exosomes and microvesicles (MVs) are small membrane-enclosed particles of endosomal and plasma membrane origin, respectively, that are released by cells into the extracellular environment. These membrane-bound vesicles represent an important mode of intercellular communication. miRNA and other “cargo” from intracellular compartments, expressed in the circulation, have been reported to be contained within EVs (reviewed in Couto et al. [34]). It has been suggested that circulating EVs can be used as noninvasive biomarkers for early detection, diagnosis, and treatment of cancer [35]. Our analysis on plasma, so far, used methods that did not distinguish between mRNA contained within or outside of EVs. It was therefore of interest to determine if the circulating transcripts are located within EVs or circulate as membrane-free molecules (EV-free).

**Figure 8:**
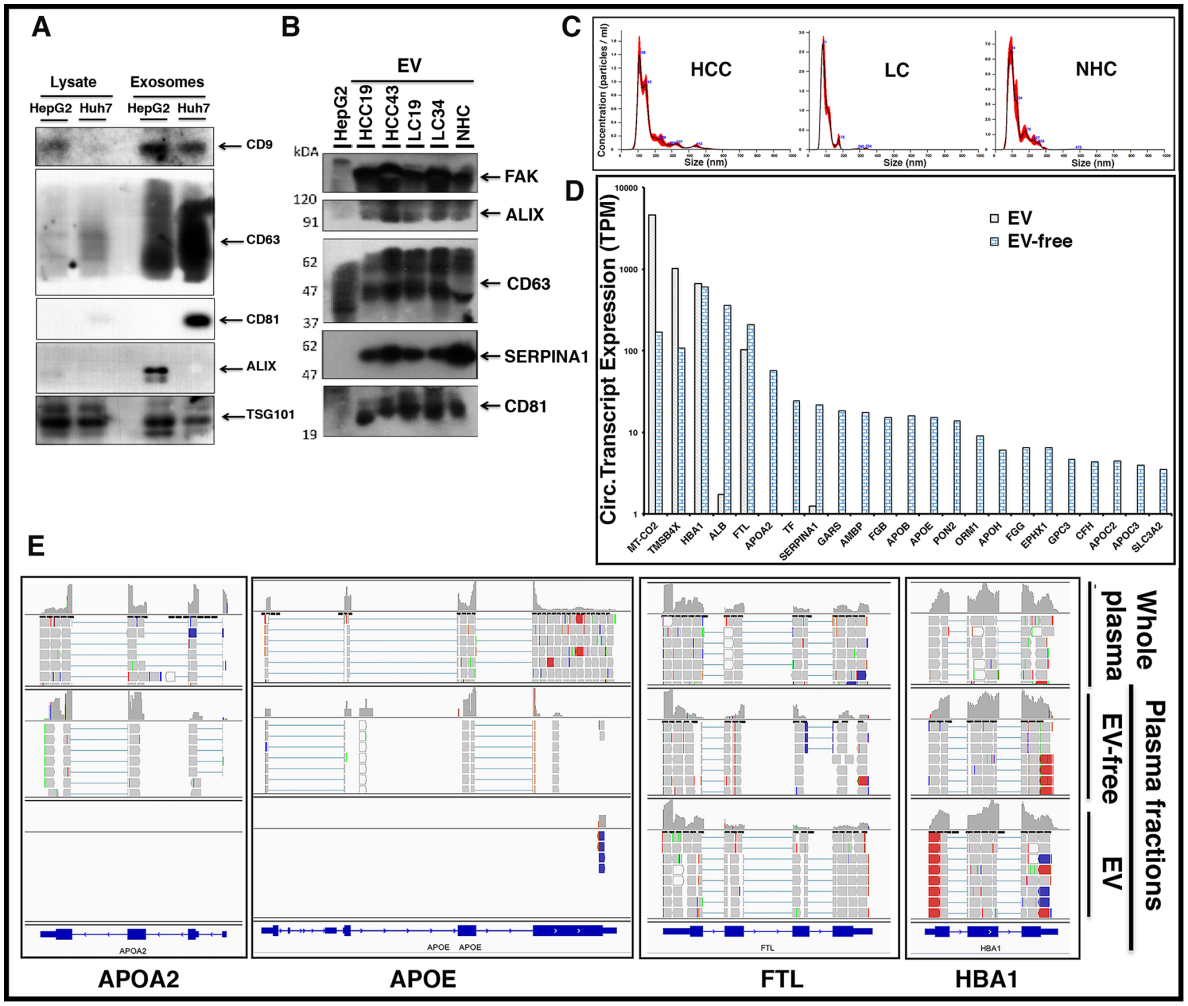
Circulating liver derived transcripts predominantly exist outside extracellular vesicles (EVs) in the plasma of HCC patients. (**A**) EVs were isolated from the culture media of HepG2 and Huh7 liver cancer cells and characterized by western blotting. Equal amounts of protein samples were loaded and the enrichment of tetraspanin receptors CD9, CD63, CD81, Alix, and TSG101 was tested as markers of EVs. (**B**) EVs from either HCC, LC, or normal healthy control (NHC) plasma samples were extracted using ExoQuick kit and further purified using ultracentrifugation. In addition to EV markers Alix, CD63, and CD81, liver specific marker SERPINA1 was investigated. FAK was used as a loading control. (**C**) Characterization of EVs from various plasma samples was done by NTA NanoSight NS300 system and NTA software analysis was used to obtain the size distribution and quantitation of particles. (**D**) Plasma sample from an HCC patient (HCC101P) was fractionated into EV fraction and remaining EV-free fraction. RNAseq analysis was carried out on RNA from total plasma and its two fractions. Comparative expression profiles (TPM) of circulating transcripts within and outside EVs for more than 20 liver derived transcripts are shown. (**E**) Read piles of some of the liver specific transcripts shown above in 6D for whole patient plasma, EVs, and EV-free fraction highlight the differential expression. *FTL* and *HBA1* show almost equivalent levels of transcripts within and outside EVs, but *APOA2* and *APOE* preferentially exist outside EVs.

To optimize the purification of EVs in plasma samples, we first evaluated EV isolation from liver cancer cell lines. Figure 8A shows the enrichment of unique EV markers CD9, CD63, CD81, and Alix by immunoblotting. EV isolation was then carried out with HCC and LC patient plasma samples and further characterization carried out by nanoparticle tracking analysis (NTA) and immunoblotting (Figure 8B, 8C; Supplementary Figure 4). NTA of these samples confirmed particle size ranges between 50 to 150 μm, which are specifically characteristic of exosomes (Figure 8C). RNA was isolated from total plasma and two plasma sub-fractions, EV and EV-free, from the same patient (HCC101P) and followed by RNAseq analysis. The results show that although transcripts such as *MT-CO2*, *FTL*, *TMSB4X*, *HBA1*, and other mitochondrial and ribosomal transcripts were uniformly present inside and outside circulating EVs, liver specific transcripts were predominantly abundant in the EV-free fraction. Bar graphs (Figure 8D) highlight the differential expression (TPM) of circulating transcripts. The results suggest that major tissue specific transcripts are predominantly present in plasma outside vesicles. Figure 8E shows a snapshot of read piles of four circulating transcripts including liver specific *APOA2* and *SERPINA1* in EV/EV-free fractions and total unfractionated plasma. Note that *FTL* and *HBA1* transcripts are represented in both fractions, while *APOA2* and *APOE* are preferentially detected in EV-free plasma, highlighting the differential expression. It is tempting to speculate that these transcript fragments might exist as protein nucleic acid complexes that ensure protection from ubiquitous nucleases in the circulation.

## DISCUSSION

Here we show that human plasma, among a diverse family of RNA transcripts, contains mRNA corresponding to more than 19,000 different coding genes. The differential sequencing depths and read pile densities within exons suggest that circulating mRNA transcripts likely exist as fragments. Investigation into the contents of circulating EVs implied that mRNA transcripts are located within and outside EVs. Many of these coding transcripts came from the liver and other solid organs, and liver derived transcripts were predominantly found outside circulating EVs. One unique observation is that the level of any circulating mRNA transcript was very similar from patient to patient, not varying by more than 8-fold for 94% of the different gene transcripts, which suggests that the circulating human mRNA transcriptome is remarkably consistent.

We demonstrate the upregulation of cancer specific transcripts in the circulation of HCC patients compared to LC patients. Consistent with our results, a growing body of evidence exists supporting the presence of circulating mRNA in various cancer patients [11, 12, 36]. Serum *hTERT* mRNA in HCC patients correlated with clinical variables such as tumor size, number, and degree of differentiation [14]. A multi-centered trial demonstrated the sensitivity/specificity of HCC diagnosis by *hTERT* mRNA as 90.2%/85.4% and indicated that it was superior to AFP, AFP-L3, and DCP in the diagnosis of HCC [15]. Similar performance using serum *hTERT* mRNA was demonstrated in breast cancer [17, 37]. Albumin mRNA has also been detected in the peripheral blood of advanced stage (TNM stages III and IV) HCC patients. While it has been reported that liver transplantation in HCC patients reduced albumin mRNA below detectable limit, recurrence of HCC led to detectable levels of circulating albumin mRNA. No circulating albumin mRNA was detected in healthy individuals or cirrhotic liver patients [38]. Higher levels of plasma albumin mRNA were also detected in LC and active hepatitis B patients with a diagnostic sensitivity of more than 85% and specificity for detection over 90% [39]. Together, these reports clearly highlight the potential of circulating RNA in the detection of HCC.

Although the numbers of HCC/LC patients represented in our study are too small for conclusive statements to be made about the prevalence or correlation of specific transcripts with any clinical states, we observed striking trends. Plasma samples from HCC patients contained elevated amounts of specific mRNAs and these samples were “grouped together” by the unsupervised analysis of the RNAseq data. Since the genes and polypeptides for many of these mRNAs (such as *ALB*, *DUSP*, *FAS*, *SDF2L1*, *STRA13*, *MGAT2*) have been reported elsewhere in the literature to be associated with HCC [15, 20, 29, 40], it is tempting to suggest that their presence as mRNA in the circulation is related to the cancer diagnosis, despite the small numbers of samples used in this study.

**Figure 9:**
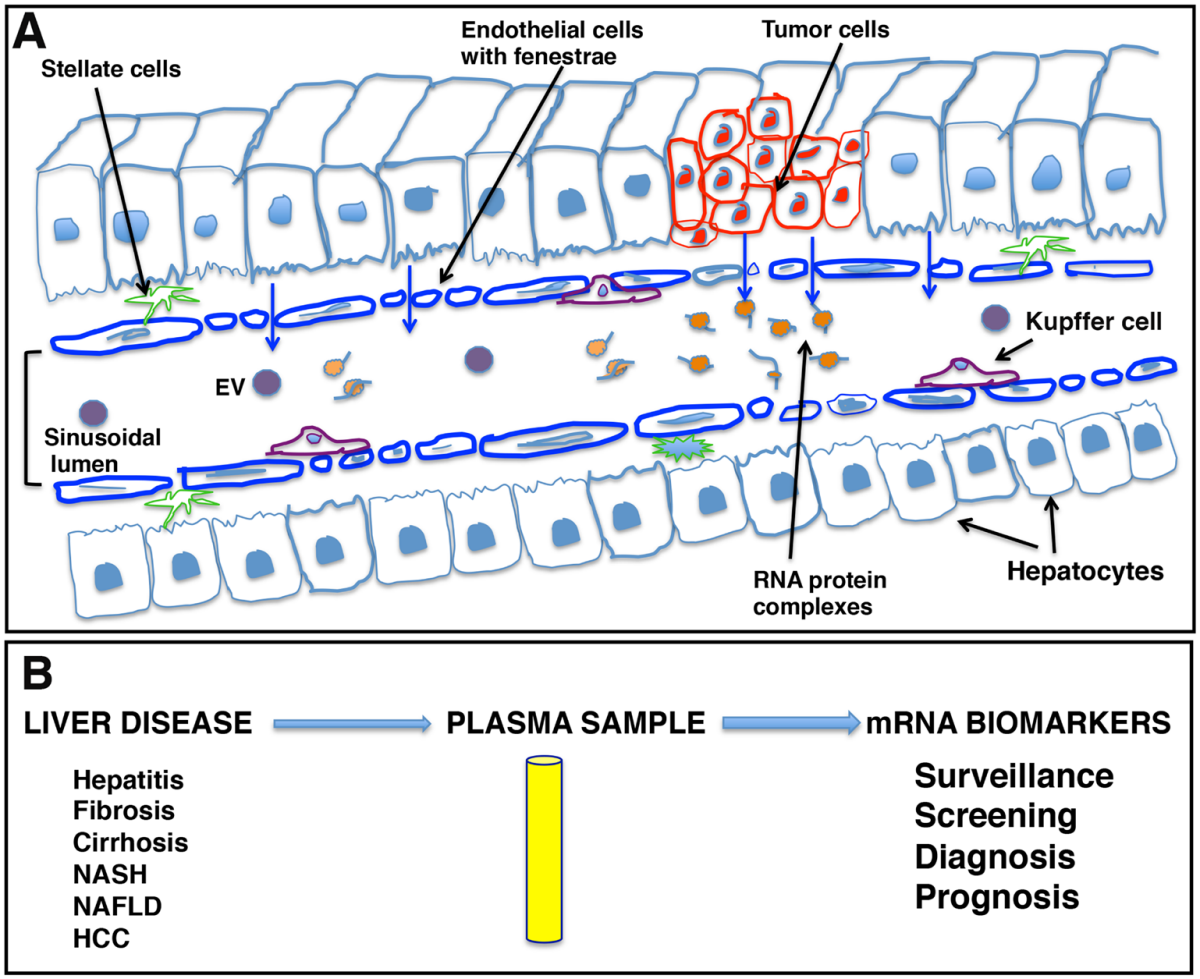
Schematic representation of liver sinusoids and loose architecture of liver endothelium. (**A**) Liver sinusoids. Sinusoids are highly modified leaky fenestrated capillaries with large lumens found in the liver, bone marrow, lymphoid tissue, and in some endocrine organs. The discontinuous architecture and fusion of the luminal and abluminal plasma membrane at fenestrae or gaps of liver sinusoidal endothelial cells (LSECs) result in leaky endothelium. This leaky vasculature may contribute to the transport of cellular cargo, extracellular vesicles (EVs), and large molecules like RNA-protein complexes from liver cells into blood. (**B**) mRNA signatures as potential liver disease biomarkers. The leaky endothelium provides an opportunity for development of a liquid biopsy platform for liver diseases, including hepatocellular carcinoma, where patient plasma can be tested for the presence of certain disease specific transcripts as biomarker analytes and can be effectively used in patient management.

Our data indicate that liver derived transcripts are predominantly present outside EVs. Endothelial cells in many blood vessels form an uninterrupted vasculature. However, as illustrated in Figure 9A, the discontinuous architecture and fusion of the luminal and abluminal plasma membrane at fenestrae, also called ‘gaps,’ of liver sinusoidal endothelial cells (LSECs) constitute a permeable barrier between blood cells on one side and hepatocytes and hepatic stellate cells on the other side, making them the most permeable endothelial cells of the mammalian body [41]. Further, the endothelium is thickened in HCC and loses its fenestrations while a discontinuous basement membrane is formed, resulting in vessels that are structurally and functionally abnormal [42]. It could be speculated that due to this known leaky vasculature in liver and other endocrine organs, detection of tissue specific circulating transcripts is not surprising.

The ability to detect mRNA in the blood could offer another non-invasive biomarker analyte and a liquid biopsy platform to detect and risk stratify disease, providing advantages to current non-invasive methods (Figure 9B). Primarily, it might be possible to detect mRNAs corresponding to non-secretory polypeptides in the blood or gene products that are present in extremely low abundance and cannot be detected with antibody assays. Also, detection of mutant genes and gene products is possible and, unlike mutated DNA that is usually single or limited copy number per cell, RNA is amplified. We have investigated the circulating human mRNA transcriptome and demonstrated dysregulation of certain liver and cancer specific mRNA transcripts in the circulation of HCC patients. However, more work is needed to determine biological significance and establish a biomarker value of mRNA signatures in HCC clinical management.

## MATERIALS AND METHODS

### Human subjects

Human plasma samples used in this study were from the Corporal Michael J. Crescenz VA Hospital, Philadelphia, PA (CMCVAMC); the University of Texas, Houston, TX; and a commercial supplier, BioChemed Services, Inc., Winchester, VA. RNAseq was carried out on plasma samples from CMCVAMC and BioChemed. Patient characteristics are summarized in Table 1 and explained in detail in Supporting Materials and Methods. RT-qPCR validation was performed on HBsAg positive HCC patients, ages 45–75 years (*n* = 10), and HBsAg/HCC/LC negative age and gender matched individual controls (*n* = 10) from University of Texas, Houston. Plasma samples and, in some cases, parallel tumor tissues from BioChemed were derived from both males and females (aged 57–74), with HCC histologically confirmed and staging provided. All samples used in our study were collected with informed consent in writing under IRB approved protocols.

### RNAseq experiment and analysis

Total RNA from 1–2 ml plasma samples (spun at 2,000g for 5 minutes) was prepared using the Qiagen RNeasy Serum/Plasma Kit (Qiagen, Valencia, CA, USA) and quantified on a NanoDrop spectrophotometer. RNAseq analysis was carried out at Cancer Genomics Facility at Thomas Jefferson University (TJU), Philadelphia, PA, USA. RNA purity and integrity was assessed by Agilent 2100 BioAnalyzer. Libraries were prepared using the SMARTer^®^ Stranded Total RNA-Seq Kit v2 (Takara Bio USA, Inc., Mountain View, CA, USA). Paired-end sequence reads were analyzed according to currently available best practices for whole-transcriptome analysis, which include alignment onto the current human reference genome assembly (GRCh38) using the STAR splice aware aligner. Transcript counts and abundances expressed in transcripts per million (TPM) were estimated based on GENCODE v28 gene annotations using the RSEM algorithm. Differential expression between groups was tested with the R/Bioconductor DESeq2 package. Tissue specific content was generated using the R/Bioconductor TissueEnrich package [43] (see Supporting Materials and Methods). Read piles of liver specific transcripts were analyzed using alignment .bam files corresponding to genome version GRCh38. The coverage density plots showed how many reads were aligned to a specific location, typically lining up with exon boundaries. Detailed information is provided in Supporting Materials and Methods. Tissue specificity of transcripts was also evaluated using Human Protein Atlas (HPA) dataset (version 19.2, Ensemble version 92.38), representing expression levels in 37 human tissues from 122 individuals based on RNAseq [44].

### RNA extraction from cell lines, tissues, extracellular vesicles (EVs), and EV-free plasma

Total RNA was extracted from HCC cell lines HepG2 and Huh7 and immortalized normal liver PH5CH cells using Qiagen miRNeasy Mini Kit (Qiagen, Valencia, CA, USA). Total RNA was also extracted from two Grade-2 HCC tumor tissues (HCC103T, HCC105T, BioChemed) and a normal liver tissue using mirVana miRNA Isolation Kit (Ambion, Austin, TX, USA). All kits were used by following manufacturer guidelines. Tumor samples corresponding to two plasma samples (HCC103P, HCC105P, BioChemed) were investigated by RNAseq and one of them (HCC103T) was also studied by RT-qPCR. 100 mg tissue treated with RNA*later*-ICE solution (Invitrogen, Carlsbad, CA, USA) was homogenized in a glass homogenizer, worked-up following kit protocol, and RNA eluted in 100 μl RNase-free water. Plasma (3 ml) from HCC101P (BioChemed) was spun at 2,000g for 15 minutes to remove cellular debris and supernatant was collected. Total RNA from extracellular vesicles (EVs) was extracted using the ExoMir Kit (BIOO Scientific Corp., Austin, TX, USA). RNA from the remaining EV-free flow-through plasma was extracted using the Qiagen RNeasy Serum/Plasma Kit (Qiagen, Valencia, CA, USA). RNA from plasma EVs, EV-free plasma, and liver tissues was sequenced at TJU as described above.

### RT-qPCR

RNA samples from cell lines and tumor tissue (2.5 μg) were subjected to DNase digestion before reverse transcription to cDNA. 50 ng qPCR reactions were set in triplicate using LightCycler 480 SYBR Green I Master reaction mix and LightCycler 480 System (Roche Diagnostics, Indianapolis, IN, USA). ΔCt values were calculated by subtracting Ct values of *GAPDH* in each sample from Ct values of target genes. ΔCt values of tumor and HCC cell lines were normalized with ΔCt values of liver PH5CH cells to yield ΔΔCt values and relative expression was calculated using the formula 2^-ΔΔCt^. RNA from clinical plasma samples (1 to 2 ml) was prepared using the Qiagen RNeasy Serum/Plasma Kit (Qiagen, Valencia, CA, USA). RNA was subjected to DNase digestion before reverse transcription to cDNA. Each cDNA sample was spiked with specific copy number of a 102 nt zebrafish-specific DNA ultramer (IDT, Coralville, IA) for use as internal control. PCR reactions for each sample (*n* = 20) with 25 ng cDNA were subjected to qPCR. Primers were designed corresponding to high sequencing depth regions identified from RNAseq (Supplementary Table 1). Relative expression of each transcript was calculated using the ΔΔCt method by normalizing with internal reference DNA ultramer and the average ΔCt value (*n* = 10) from age and gender matched NHC samples. Each PCR reaction was carried out in triplicate sets and relative expression determined.

### Semi-quantitative nested PCR and Southern blots

Clinical plasma samples from HCC patient HCC11 and NHC10 were investigated for liver derived *APOA2* mRNA. Two sets of oligos were designed. One oligo set had forward/reverse primers specific for *APOA2* exon 3/exon 4, respectively, and amplified a short product of 76 bp when no intron was present in the transcript. The other set consisted of oligos specific for the intervening intron between exons 3 and 4 and amplified an 82 bp fragment. Semi-quantitative PCR reactions were carried out using either exon 3 and 4 specific oligos or oligos corresponding to the intervening intron. PCR was carried out for 30 cycles and products were resolved by electrophoresis on a 2% agarose gel. For further validation and characterization of circulating transcripts, nested PCR in clinical plasma samples was carried out (HCC patient HCC19 and NHC18) to study *APOA2* and *FTL* transcripts. Two sets of oligos, corresponding to PCR1 extended amplicon and nested amplicon, were used to amplify *APOA2* and *FTL* cDNA. PCR1 was carried out for 30 cycles using extended PCR1 oligos. PCR1 product was diluted 12.5-fold and subjected to additional 30 cycle nested PCR using nested oligos. Both PCR1 and nested PCR products were resolved by electrophoresis on a 2% agarose gel. Outer PCR1 oligo sets yield a larger product (76 bp for *APOA2*, 113 bp for *FTL*), while inner nested oligo sets yield smaller products (47 bp for *APOA2*, 49 bp for *FTL*). PCR1 and nested PCR products were transferred to an Amersham Hybond-N^+^ membrane (GE Healthcare Life Sciences, Marlborough, MA, USA) followed by hybridization with extended PCR1 specific 5′-LICOR 800 tagged oligos (IDT, Coralville, IA, USA) for Southern blot based detection of PCR amplified transcripts. 5′-LICOR 800 probes incorporate in PCR1 products, but not in nested products.

### Isolation and characterization of EVs

EVs were purified from HepG2 and Huh7 liver cancer cell lines as previously described [45, 46] with some modifications. Briefly, the culture supernatant was collected after 48 hours of starvation, pre-cleared of any cellular debris at 2,000g for 20 minutes at 4°C, and centrifuged again at 10,000g for 30 minutes at 4°C. The supernatant was ultracentrifuged at 100,000g for 120 minutes at 4°C. The EV pellet was washed in phosphate buffered saline (PBS) at 100,000g for 120 minutes at 4°C and resuspended in PBS. Plasma samples (1 to 1.5 ml) from HCC patients and normal healthy controls (CMCVAMC) were spun at 2,000g for 30 minutes to remove cellular debris before purification of EVs using ExoQuick kit (System Biosciences, Palo Alto, CA, USA). EVs were further purified by ultracentrifugation using PBS at 100,000g for 120 minutes at 4°C. EV pellet was resuspended in PBS and quantified using NanoSight Tracking analysis (Malvern Instruments, Malvern, UK). Remaining EV suspension was lysed for further characterization. Additional details are in Supporting Materials and Methods.

### Immunoblotting (IB)

EV isolation and characterization by IB was carried out as reported earlier [47, 48]. Blocked membranes were probed with either rabbit monoclonal antibodies (mAbs) to TSG101 (ab125011) from Abcam (Cambridge, UK) and CD63 (55051) and FAK (13009) from Cell Signaling Technologies (Danvers, MA, USA) or mouse mAbs to human CD81 (ab23505) from Abcam, Alix (sc53540) and human CD9 (sc13118) from Santa Cruz Biotechnology, Inc. (Dallas, TX, USA), and human SERPINA1 (NBP2-52557) from Novus Biologicals (Littleton, CO, USA).

### Statistical analysis

The reads for each mRNA obtained from the plasma of HCC, LC, and NHC patients were analyzed in separate generalized linear models with the assumption of negative binomial (NB) distribution using the DESeq2 software package. The *p*-values for each set of tests were adjusted for multiple testing using the False Discovery Rate (FDR) of 1%. RT-qPCR data are shown as mean values and considered statistically significant at *p* < 0.05. GraphPad Prism software (San Diego, CA, USA) was used for analysis. Western and Southern blot figures were assembled in Adobe Photoshop (Adobe Inc., San Jose, CA, USA).

## SUPPLEMENTARY MATERIALS



## Abbreviations

HCC: Hepatocellular carcinoma
LC: Liver cirrhosis
miRNA: microRNA
mRNA: messenger RNA
RNAseq: RNA sequencing
RT-qPCR: Reverse transcription-quantitative polymerase chain reaction
NHC: Normal healthy control
EVs: Extracellular vesicles
MVs: Microvesicles
