# Supplementary material for: Profiling the circulating mRNA transcriptome in human liver disease
Source: Oncotarget. 2020 Jun 9;11(23):2216–32. doi: 10.18632/oncotarget.27617 (PMC7289528; doi:10.18632/oncotarget.27617)
Supplement: Supplementary file 1 [file oncotarget-11-2216-s001.pdf]

Profiling the circulating mRNA transcriptome in human liver disease

SUPPLEMENTARY MATERIALS

Human subjects and specimen

Human plasma samples used in this study were from the Corporal Michael J. Crescenz VA Hospital, Philadelphia, PA, USA (CMCVAMC); the University of Texas, Houston, TX, USA; and a commercial supplier, BioChemed, Inc., Winchester, VA, USA. RNAseq was carried out on plasma samples from CMCVAMC and BioChemed Services, Inc., while RT-qPCR validation was performed using the samples from the University of Texas. As summarized in Table 1, samples from CMCVAMC were from individuals with ages ranging from 54 to 63 years, were all male, and most had cirrhosis due to chronic untreated hepatitis C virus (HCV) infection that was confirmed clinically, radiologically, or histologically. Diagnosis of HCC of the 10 cases from the University of Texas was based on histopathology and/or 2 imaging modalities (i.e. MRI and CT/US). Diagnosis of LC was based on liver pathology and/or clinical lab and imaging findings of hepatic decompensation or portal hypertension. Plasma samples and in some cases parallel tumor tissues from BioChemed were derived from both males and females (aged 57–74), where HCC was histologically confirmed and staging provided. Plasma samples from University of Texas were comprised of HBsAg positive HCC patients (n = 10) and HBsAg negative, HCC/LC negative individual controls (n = 10), ages 45–75 years. All samples used in our study were collected with informed consent in writing under IRB approved protocols.

RNAseq experiments

Total RNA from 1–2 ml plasma samples (sedimented at 2,000g for 5 min) was prepared using the Qiagen RNeasy Serum/Plasma Kit (Qiagen, Valencia, CA, USA) and quantified on a NanoDrop One spectrophotometer utilizing A260/A280 ratios to assess nucleic acid purity. RNAseq analysis was carried out at Cancer Genomics Facility at Thomas Jefferson University, Philadelphia, PA. RNA purity and integrity were assessed by Agilent 2100 BioAnalyzer and RIN values were determined. Libraries were prepared using the SMARTer® Stranded Total RNA-Seq Kit v2 (Takara Bio USA, Inc., Mountain View, CA, USA) to support library preparation from as little as 250 pg of RNA. The protocol used was for preparation of total stranded RNA with ribosomal RNA and mitochondrial RNA targeted for removal. Quality of each library was assessed with the Agilent TapeStation 2200. Six to eight libraries were pooled (n = 6–8) and sequenced using Illumina v2 chemistry, 2 × 75 base paired-reads, on one high-output flow cell of an Illumina NextSeq 500 to achieve at least 50 million paired reads per sample in 75-base read length paired-end mode. Adapter trimming was performed on the resulting paired end fastq files, followed by trimming residual Pico v2 SMART Adapters by removing the first 3 nucleotides from the second read in each pair.

RNAseq data analysis

Paired-end sequence reads were analyzed according to currently available best practices for whole-transcriptome analysis, which include alignment onto the current human reference genome assembly (GRCh38) using the STAR splice aware aligner. Transcript counts and abundances expressed in transcripts per million (TPM) were estimated based on GENCODE v28 gene annotations using the RSEM algorithm. Differential expression between groups was tested with the R/Bioconductor DESeq 2 package. Tissue specific content was generated using the R/Bioconductor TissueEnrich package. Most abundant highly expressed genes from RNAseq data were inputted to determine which tissue-specific genes are enriched in those datasets. Tissue-specific genes were defined by processing RNAseq data from the Human Protein Atlas (HPA) [1], GTEx [2], and mouse ENCODE [3] using the algorithm from the HPA. The hypergeometric test was used to determine if the tissue-specific genes are enriched among the input genes. Tissue enrichment is defined as genes with an expression level greater than 1 (TPM or FPKM) that also have at least five-fold higher expression levels in a particular tissue compared to all other tissues. Fold change in liver tissue, for example, would be calculated as: Fold change = (k/n)/(Kb/Nb), where k is the number of detected liver specific genes, n is the number of all detected genes, Kb is the number of liver specific genes in the human genome, and Nb is the number of genes in the human genome. Read piles of liver specific transcripts were analyzed using alignment .bam files corresponding to genome version GRCh38.

The coverage density plots showed how many reads are aligned to a particular location, typically lining up with exon boundaries.

RT-qPCR analysis

Total RNA from 1–2 ml plasma samples (University of Texas, Houston) was prepared using the Qiagen RNeasy Serum/Plasma Kit (Qiagen, Valencia, CA, USA) and quantified on a Nanodrop One spectrophotometer. RNA (1 µg) was subjected to DNase digestion before reverse transcription to cDNA. Each cDNA sample was spiked with a 102 nt zebrafish specific DNA ultramer (IDT, Coralville, IA) for use as internal control. A 200 pM stock of DNA ultramer was made and a serial dilutions were prepared from 20 nM to 0.02 oM and subjected to qPCR using ultramer specific oligos. The standard curve demonstrated that ultramer concentration was inversely proportional to Ct values obtained and melting curves reflected the specificity of ultramer amplification (data not shown). To validate RNAseq results, all cDNA samples were spiked with 20 pM ultramer, which produced a Ct of 14 in the standard curve (data not shown). PCR reactions for each sample ($n = 20$) with 25 ng cDNA were subjected to RT-qPCR. For effective amplification of circulating mRNA fragments, primers were designed from RNAseq high sequencing depth regions. Relative expression of each transcript was calculated using the ΔΔCt method by normalizing with internal reference DNA ultramer and the average ΔCt value ($n = 10$) from age and gender matched NHC samples. Each PCR reaction was carried out in triplicate sets and relative expression determined.

Isolation and characterization of EVs

EVs were purified from HepG2 and Huh7 liver cancer cell lines as previously described [4–6] with some modifications. Briefly, the culture supernatant was collected after 48 hours of starvation. The supernatant was first pre-cleared of any cellular debris by centrifugation at 2,000g for 20 minutes at 4°C and subsequently transferred to a fresh ultracentrifuge tube and centrifuged at 10,000g for 30 minutes at 4°C. The remaining supernatant was centrifuged to purify the EV fraction at 100,000g for 120 minutes at 4°C. The EV pellet was washed by a second centrifugation in 1X PBS at 100,000g for 120 minutes at 4°C. The final EV pellet was resuspended in 1X PBS for storage at –80°C. Plasma samples from HCC patients and normal healthy controls (CMCVAMC) were subjected to centrifugation at high speed for 30 minutes to remove residual cellular debris and supernatant collected. A minimum of 1 ml of plasma from patients was used to purify EVs by commercial ExoQuick kit (System Biosciences, Palo Alto, CA, USA) and isolated EVs were further purified by ultracentrifugation using 1X PBS at 100,000g for 120 minutes at 4°C. EV pellet was resuspended in 100 µl PBS. One µL of the EV suspension was used for NanoSight NS300 (Malvern Instruments, Malvern, UK) analysis after diluting 1:1,000 in PBS, at Thomas Jefferson University. The NTA software (Malvern Instruments, Malvern, UK) was used to obtain the size distribution and concentration of particles. Remaining EV suspension was lysed for characterization by immunoblotting using primary Abs against CD9, CD63, CD81, and TSG101 followed by secondary antibody treatments.

REFERENCES

1. Uhlén M, Fagerberg L, Hallström BM, Lindskog C, Oksvold P, Mardinoglu A, Sivertsson Å, Kampf C, Sjöstedt E, Asplund A, Olsson I, Edlund K, Lundberg E, et al. Tissue-based map of the human proteome. Science. 2015; 347:1260419. https://doi.org/10.1126/science.1260419. [PubMed]
2. GTEx Consortium. The genotype-tissue expression (GTEx) pilot analysis: multitissue gene regulation in humans. Science. 2015; 348:648–60. https://doi.org/10.1126/science.1262110. [PubMed]
3. Shen Y, Yue F, McCleary DF, Ye Z, Edsall L, Kuan S, Wagner U, Dixon J, Lee L, Lobanenkov VV, Ren B. A map of the cis-regulatory sequences in the mouse genome. Nature. 2012; 488:116–20. https://doi.org/10.1038/nature11243. [PubMed]
4. Théry C, Amigorena S, Raposo G, Clayton A. Isolation and characterization of exosomes from cell culture supernatants and biological fluids. Curr Protoc Cell Biol. 2006; 30:3.22.1–29. https://doi.org/10.1002/0471143030.cb0322s30. [PubMed]
5. Fedele C, Singh A, Zerlanko BJ, Iozzo RV, Languino LR. The αvβ6 integrin is transferred intercellularly via exosomes. J Biol Chem. 2015; 290:4545–51. https://doi.org/10.1074/jbc.C114.617662. [PubMed]
6. Singh A, Fedele C, Lu H, Nevalainen MT, Keen JH, Languino LR. Exosome-mediated transfer of αvβ3 integrin from tumorigenic to nontumorigenic cells promotes a migratory phenotype. Mol Cancer Res. 2016; 14:1136–46. https://doi.org/10.1158/1541-7786.mcr-16-0058. [PubMed]

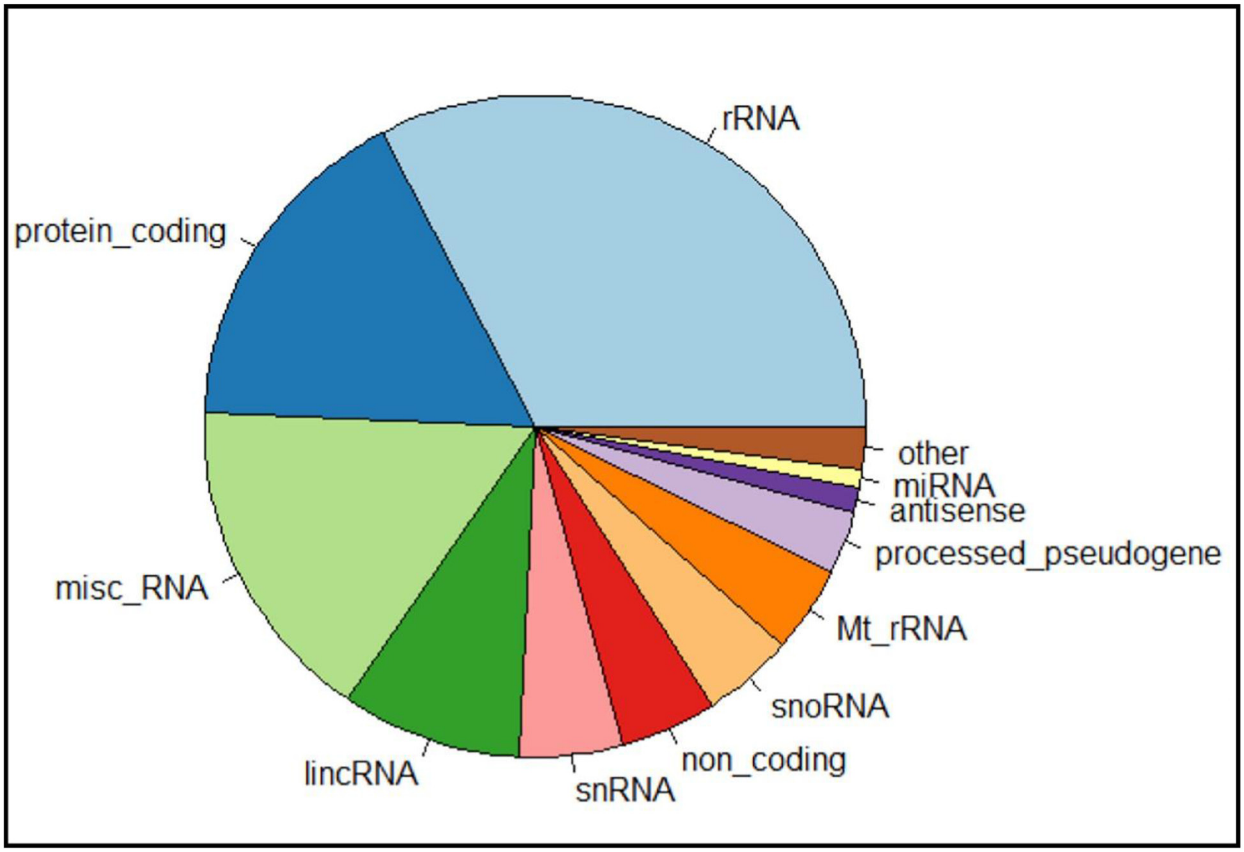

Supplementary Figure 1: Profile of RNA present in the circulation. Total RNA was extracted from human plasma samples (*n* = 21) and subjected to RNAseq analysis, as in Materials and Methods. Reads were matched to the genome databases (Materials and Methods) and the relative abundance of each transcript family (coding and non-coding) is presented as a percentage of the total detected. The pie chart represents "transcript category" across the whole transcriptome for every million transcripts detected.

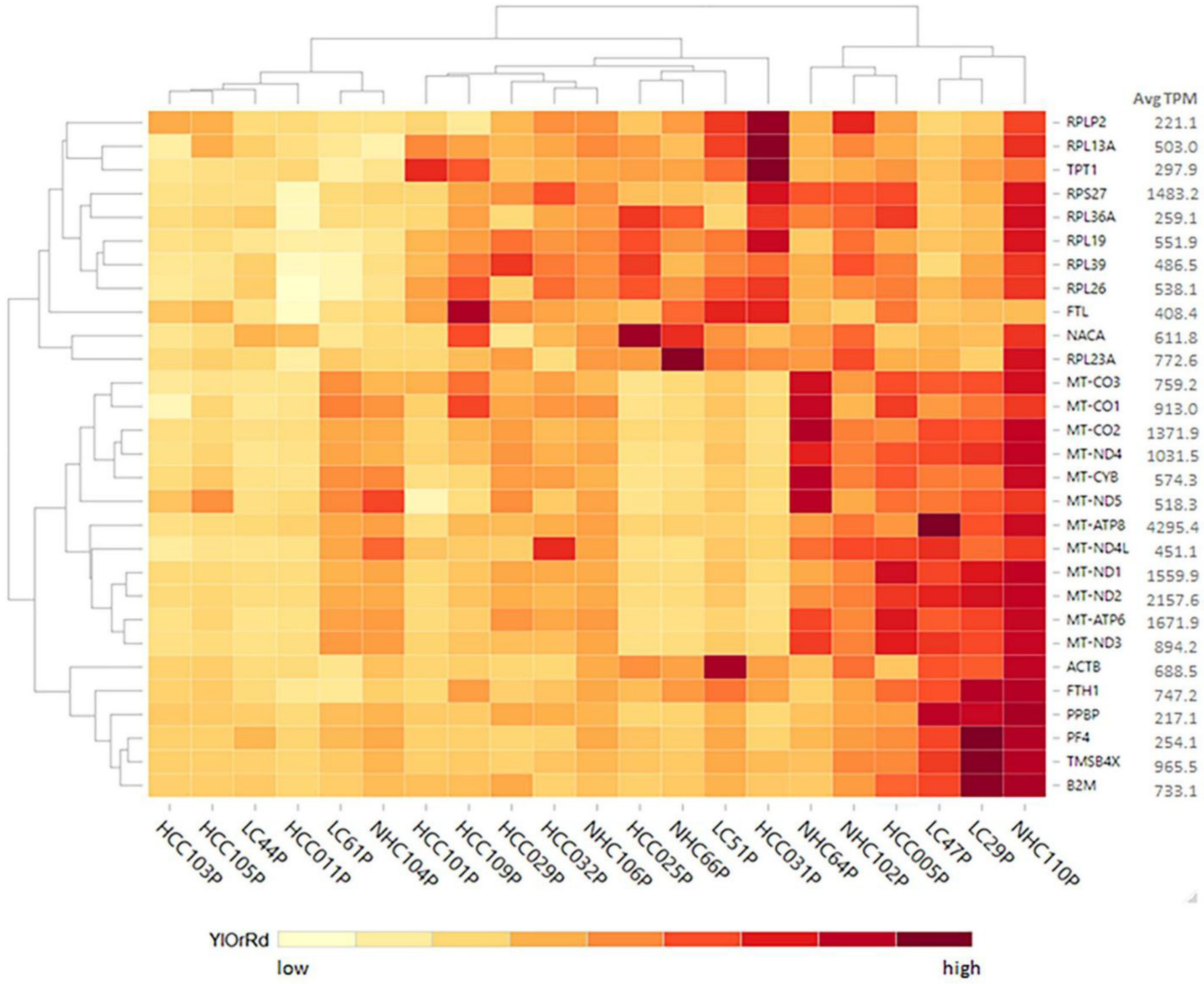

Supplementary Figure 2: Heatmap of the genes corresponding to the most abundant transcripts in plasma. The most abundant transcripts detected in plasma are shown. Columns represent patient samples with the diagnosis of the donor indicated with the prefixes NHC ("normal healthy control"); LC (liver cirrhosis); and HCC (hepatocellular carcinoma). Each row indicates the gene to which a transcript is associated. Gene nomenclature is from TissueEnrich. Numerical values in the right column represent average TPMs for a given gene for all 21 samples. Supervised hierarchical clustering where the genes were first sorted on the basis of transcript abundance (TPM) and the profiles of the 29 most abundant transcripts are shown.

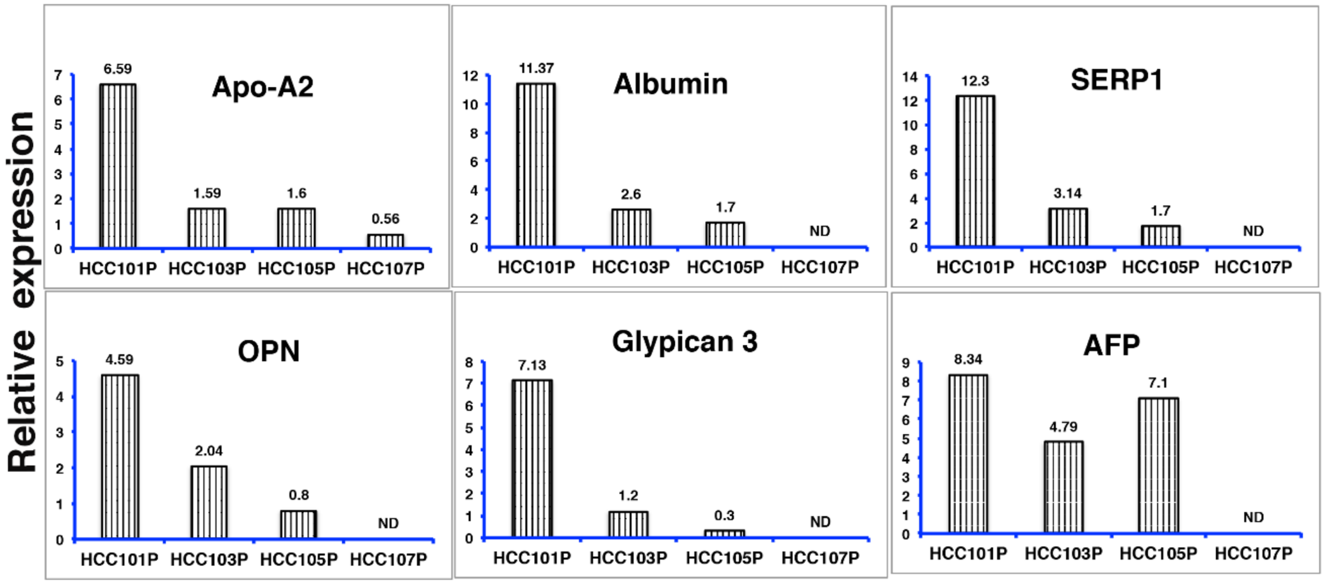

Supplementary Figure 3: RT-qPCR analysis of prominent liver derived transcripts in some plasma samples studied by RNAseq. Total RNA from 1 ml plasma samples of HCC patients (*n* = 4) and normal healthy controls (*n* = 4) prepared using the Qiagen RNeasy Serum/Plasma Kit (Qiagen, Valencia, CA, USA). One µg RNA was subjected to DNase digestion before RT reaction to cDNA. An artificial miRNA UniSP6 (Exiqon Qiagen, Germantown, MD, USA) was spiked in cDNA samples before qPCR to serve as an internal reference and provide a reference Ct value. 50 ng cDNA were subjected to real time PCR. Relative expression of each transcript was calculated using ΔΔCt method by normalizing with internal reference and non-HCC age and gender matched controls. Each PCR reaction was carried out at least in triplicate sets and relative expression determined for each transcript. ND: Ct values could not be determined.

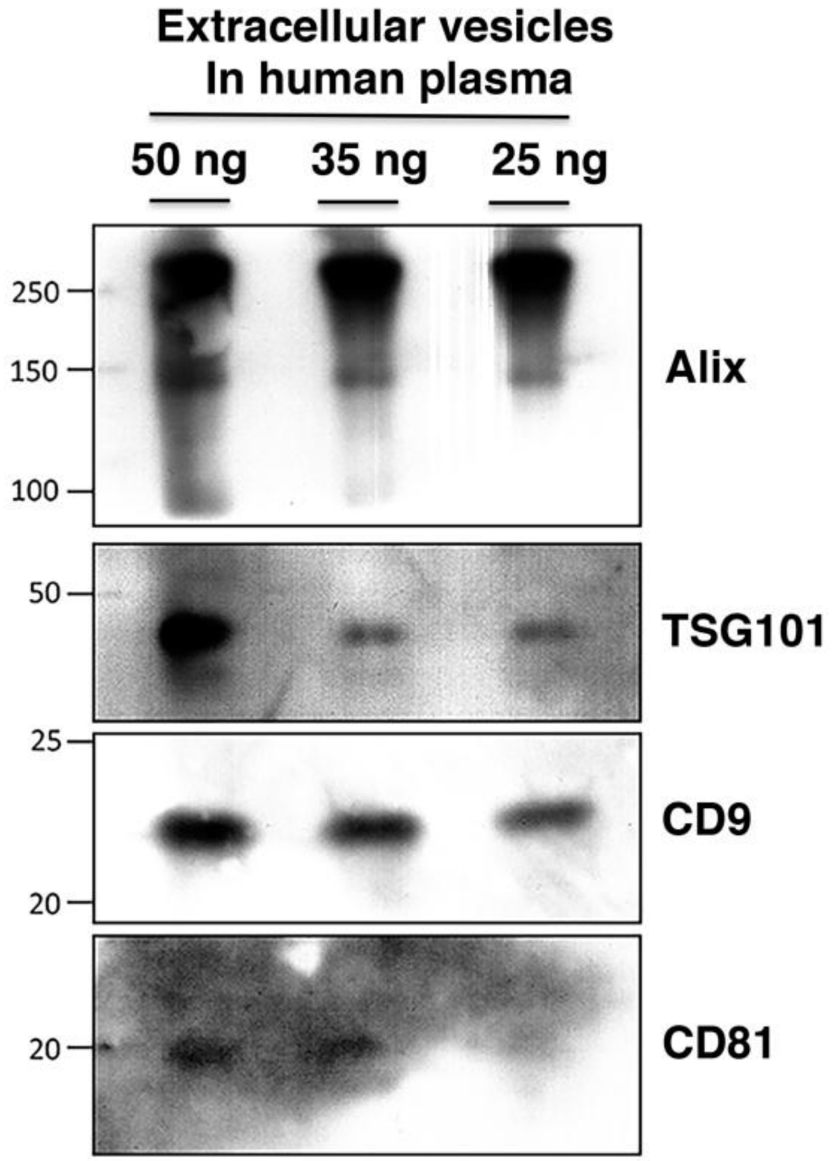

Supplementary Figure 4: Western blot demonstrating the isolation of extracellular vesicles (EVs) from human plasma. EVs were isolated from 10 ml plasma from normal healthy individual by differential ultracentrifugation as described in Materials and Methods. EVs were resuspended in 1X PBS and proteins quantitated using Pierce BCA Protein Assay Kit (Thermo Fisher Scientific, Waltham, MA, USA). Different amounts of EV protein extract, 25, 35 and 50 ng, were loaded on SDS-PAGE gel and analyzed by immunoblotting. Typical EV markers Alix, TSG101, CD9, and CD81 were evaluated using specific antibodies. We demonstrate the purity of EVs isolated from human plasma sample.

Supplementary Table 1: Oligos used in RT-qPCR, semi-quantitative PCR, and Southern blot analyses of circulating transcripts of plasma from HCC and NHC patients

Target	5′Forward3′	5′Reverse3′
ALBUMIN	CCTGATTACTCTGTCGTGCT	TTGGCATAGCATTCATGAGGAT
APOA2	GTGTCAGCTGCTCCTTTGACT	ATGGAGAAGGTCAAGAGCCC
APOA2 LICOR 800	GTGTCAGCTGCTCCTTTGACT	-
APOA2 Nested	GGTCAAGAGCCCAGAGCTTCA	TTTCAAAGTAAGACTTGGCCTCGG
APOH	CAGGACTGTGGCCCATCAAC	TCGTATAGCGTACGGCTCCAT
CYP2E1	ACTCTGAGATATGGGCTCCTGA	TATCCTTGATGGCAGGGATTCG
FGL1	GATCAGTCTGGCTGGTGGTT	TGCCAGGTGTACCAGACAATC
FTL	CGGGTCTGTCTCTTGCTTCA	GAAGATGGTCCCGGAGGTTG
FTL LICOR 800	CGGGTCTGTCTCTTGCTTCA	-
FTL Nested	TCCGGGGACTCTCTTCCAG	GCGGAGAGGAAATCGGAGG
GAPDH	AAGGTCGGAGTCAACGGATTTG	CCATGGGTGGAATCATATTGGAA
HP	TGTCATTGCCCTCCTGCTCT	ATTGCCTGAGTCCACTGCAA
HPX	GGGAGTTCAACTGGTCACCCA	CATAGGCTCCACAACCCCAG
MT-CO1	CCAATACCAAACGCCCCTCT	TGTTGAGGTTGCGGTCTGTT
SAA2	TGGCAGCATCATAGTTCCCC	TGTGGAGAGCCTACTCTGAC
SERPINA1	GTCAAGGACACCGAGGAAGAG	CCCTCATCAGGCAGGAAGAA
TRANSFERRIN	CAAGCCTGTGAAGTGGTGTG	GATGCAGTCTTCGGTGGTCT
DNA ULTRAMER	GCCGAATTTCACGGCTCATC	GTAGCCGGTCAAAGTGTCCC
